# Predictive Potential of Flow Cytometry Crossmatching in Deceased Donor Kidney Transplant Recipients Subjected to Peritransplant Desensitization

**DOI:** 10.3389/fmed.2021.780636

**Published:** 2021-12-14

**Authors:** Klara Osickova, Petra Hruba, Katerina Kabrtova, Jiri Klema, Jana Maluskova, Antonij Slavcev, Janka Slatinska, Tomas Marada, Georg A. Böhmig, Ondrej Viklicky

**Affiliations:** ^1^Department of Nephrology, Institute for Clinical and Experimental Medicine, Prague, Czechia; ^2^Transplant Laboratory, Institute for Clinical and Experimental Medicine, Prague, Czechia; ^3^Department of Immunogenetics, Institute for Clinical and Experimental Medicine, Prague, Czechia; ^4^Department of Computer Science, Faculty of Electrical Engineering, Czech Technical University, Prague, Czechia; ^5^Department of Pathology, Institute for Clinical and Experimental Medicine, Prague, Czechia; ^6^Aesculab Pathology, Prague, Czechia; ^7^Department of Transplant Surgery, Institute for Clinical and Experimental Medicine, Prague, Czechia; ^8^Division of Nephrology and Dialysis, Department of Medicine III, Medical University Vienna, Vienna, Austria

**Keywords:** kidney, transplantation-kidney, HLA incompatibility, donor specific antibodies, rejection, flow cytometry, immunosuppression

## Abstract

Recipient sensitization is a major risk factor of antibody-mediated rejection (ABMR) and inferior graft survival. The predictive effect of solid-phase human leukocyte antigen antibody testing and flow cytometry crossmatch (FCXM) in the era of peritransplant desensitization remains poorly understood. This observational retrospective single-center study with 108 donor-specific antibody (DSA)-positive deceased donor kidney allograft recipients who had undergone peritransplant desensitization aimed to analyze variables affecting graft outcome. ABMR rates were highest among patients with positive pretransplant FCXM vs. FCXM-negative (76 vs. 18.7%, *p* < 0.001) and with donor-specific antibody mean fluorescence intensity (DSA MFI) > 5,000 vs. <5,000 (54.5 vs. 28%, *p* = 0.01) despite desensitization. In univariable Cox regression, FCXM positivity, retransplantation, recipient gender, immunodominant DSA MFI, DSA number, and peak panel reactive antibodies were found to be associated with ABMR occurrence. In multivariable Cox regression adjusted for desensitization treatment (AUC = 0.810), only FCXM positivity (HR = 4.6, *p* = 0.001) and DSA number (HR = 1.47, *p* = 0.039) remained significant. In conclusion, our data suggest that pretransplant FCXM and DSA number, but not DSA MFI, are independent predictors of ABMR in patients who received peritransplant desensitization.

## Introduction

Preformed antibodies directed against donor human leukocyte antigen (HLA) antigens represent a major obstacle in kidney transplantation, limiting both access to transplantation and kidney allograft survival (1, 2). It is widely accepted that kidney transplantation across donor-specific antibodies (DSA) identified either by solid-phase assays or flow cytometry crossmatch (FCXM) is associated with a higher risk of antibody-mediated rejection (ABMR) and inferior allograft outcomes, even in absence of positive complement-dependent cytotoxicity crossmatch (complement-dependent cytotoxicity crossmatch [CDC XM]) (3–7). Several transplant programs have implemented peritransplant desensitization regimens using T- and B-cell depleting antibody induction, peritransplant apheresis, and high-dose intravenous immunoglobulin (IVIg) to counteract the deleterious effects of preformed DSA (8). Despite intense strategies of desensitization, there is still an increased rejection risk, which may critically depend on the strength of the preformed DSA. Previously, the Viennese group used anti-thymocyte globulin (ATG) induction and peritransplant immunoadsorption (IA) as desensitization regimens in DSA-positive deceased donor kidney transplantation. The only predictor of antibody-mediated rejection found by this study was donor-specific antibody mean fluorescence intensity (DSA MFI) (9). FCXM may have several advantages over DSA MFI in terms of better predictive power to select grafts at risk of ABMR (10, 11). Moreover, kidney transplantation with a low level of DSA with or without a low positive B-cell FCXM was found to be associated with satisfactory outcomes in highly sensitized mostly living donor kidney transplant recipients who received depleting antibody induction and frequently also desensitization (12). In HLA incompatible deceased donor kidney transplantation with peritransplant desensitization, outcome predictors are poorly understood. Therefore, in this retrospective single-center observational cohort study, we assessed several variables to predict antibody-mediated rejection in those DSA positive deceased donor kidney transplant recipients who had undergone peritransplant desensitization.

## Patients and Methods

### Study Design and Population

**Figure 1:**
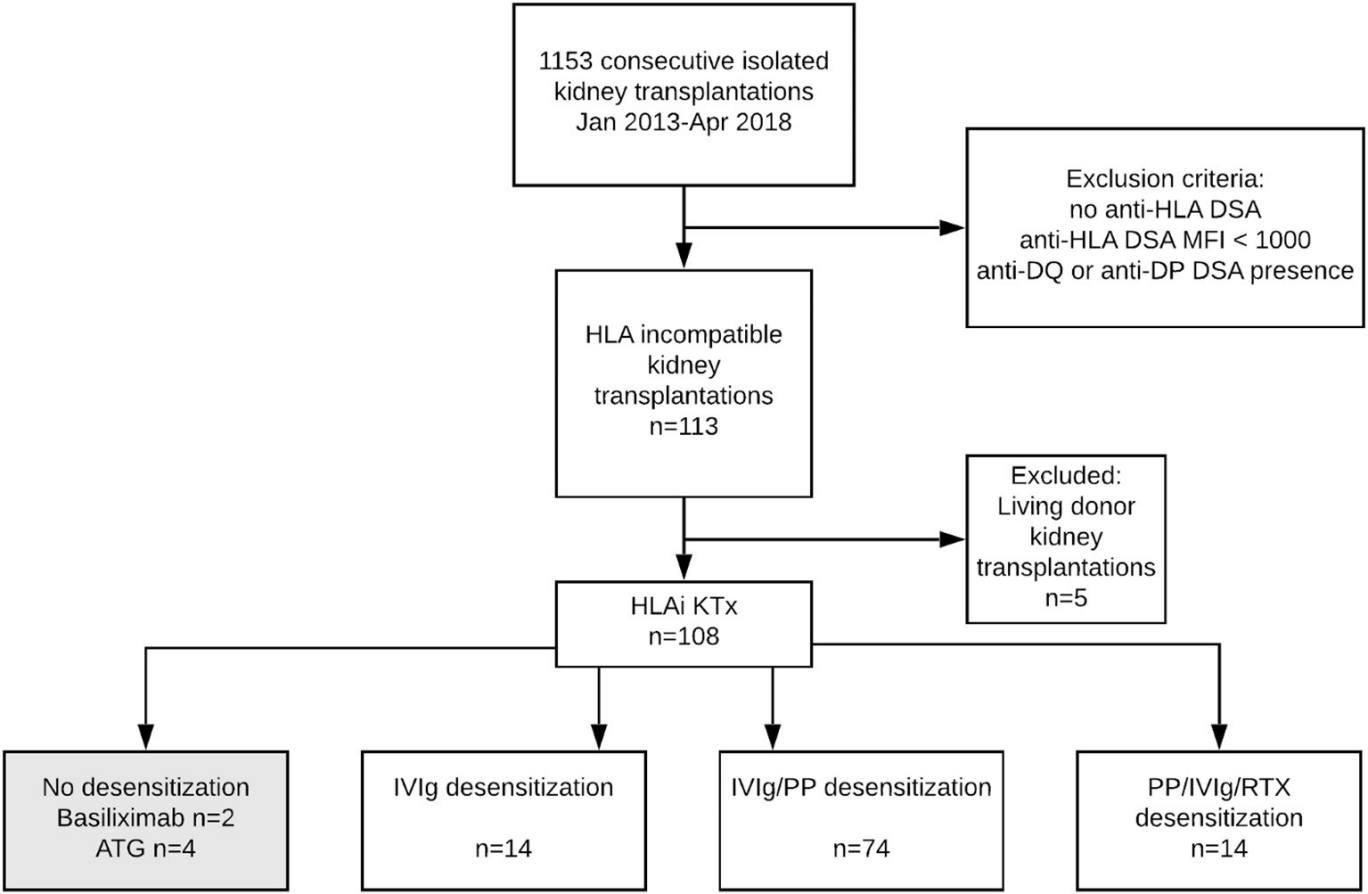
Flow diagram of kidney transplant recipients included in the study and study cohort's definition. DSA, Donor-specific Antibodies; HLA, Human Leukocyte Antigen; KTx, Kidney Transplantation; ATG, Antithymocyte Globulin; IVIg, Intravenous Immunoglobulin; PP, Plasmapheresis; RTX, Rituximab.

Our study was a single-center, cohort observational study with retrospective data analysis. A study flow chart is shown in Figure 1. All patients who underwent kidney transplantation from a deceased donor between January 2013 and April 2018 and had a positive DSA were included. Out of 1,153 allograft recipients who received a kidney transplant during the study period, 360 (31%) subjects had preexisting anti-HLA antibodies, and among those, 113 (9.8%) had one or more preformed DSA detected by solid-phase testing. The presence of circulating anti-HLA-A, -B, -C, -DR, and -DQ antibodies were annually screened using solid-phase testing. The arbitrary threshold for positivity was defined as 1000 MFI. Therefore, anti-HLA antibody specificities were accessible at the time of the transplant offer, and donor-specific antibodies were identified before transplantation based on historical Luminex assessment. Patients with DSA > 1,000 MFI and a negative current complement-dependent cytotoxicity crossmatch (CDC XM) were included (Figure 1). Immunodominant DSA was defined as the highest MFI from the last available pretransplant sera. Due to missing donor DQ typing, the anti-DQ antibodies were not traced as DSA and, therefore, excluded from the analysis. Patients were followed until the allograft loss or end of the follow-up; the median follow-up was 1,110 days. Histological diagnosis of ABMR was defined according to the latest Banff criteria (13). The design of this retrospective observational study was approved by The Ethics Committee of the Institute for Clinical and Experimental Medicine and Thomayer Hospital while ensuring the anonymity and confidentiality of the data (listed as No. A-19-24).

### Immunosuppression and Desensitization

All patients initially received triple-drug maintenance immunosuppression based on tacrolimus (Advagraf, Astellas, 0.2 mg/kg/day, target trough levels within the first 14 days at 8–15 ng/ml), mycophenolate mofetil (Cellcept, Roche, 2 g/day or generics) or enteric-coated mycophenolic acid (Myfortic, Novartis, 1440 mg/day), and tapered prednisone (initial dose 20 mg tapered to 5 mg at 3 months). All but two patients received rabbit anti-thymocyte globulin (Thymoglobuline, Genzyme, first dose of 1.5 mg/kg initiated before reperfusion, a total cumulative dose aimed at 5–7 mg/kg) induction immunosuppression. Patients received desensitization protocol mostly with plasmapheresis and IVIg, patients at highest risk received rituximab in addition. Details on desensitization strategies are given in Figure 1, **Table 3**. Further analyses were adjusted for desensitization strategy to account for the heterogeneity of applied treatments.

Recipients diagnosed with ABMR were treated with high dose steroids, plasmapheresis (1 plasma volume; 5–10 sessions per patient), and IVIg administration (0.5 g/kg) after each session. In the cases of refractory ABMR, bortezomib (Velcade, Johnson & Johnson) was administered as previously described in detail (11).

### Pretransplant FCXM and Luminex

Donor splenocytes (50 μl, 5 × 10^4^ cells) were incubated with patient or negative control sera (50 μl) for 30 min at 21°C. After washing three times in PBS (400 × *g*, 5 min), samples were incubated at 21°C for 30 min in an antibody cocktail – 5 μl anti-CD45-KO (Beckman Coulter, IN, USA), 5 μl anti-CD3-PE (Becton Dickinson, SJ, USA), 5 μl anti-CD19-PC5 (Beckman Coulter, IN, USA), and goat anti-human IgG-FITC (Jackson ImmunoResearch, West Grove, PA, USA), again washed, fixed (Cellfix), and measured on a Navios flow cytometer (Beckman Coulter, IN, USA). Fluorescein (FITC) fluorescence of patient samples was compared with the mean fluorescence of negative control samples. Cut off point was calculated as the ratio between the mean fluorescence intensity of samples and negative control sera. Cut off for T cells was 2 and for B cells was 2.5, respectively.

The FCXM positivity was defined as T-cell and/or B-cell FCXM positivity. The serum used for FCXM was obtained immediately pretransplant and was used for CDC XM.

The specificity of HLA antibodies was defined by LABScreen Mixed and Single Antigen (SAB) class I and class II beads (OneLambda Inc., CA, USA). The assay was performed according to the manufacturer's protocol. Samples were analyzed by the LabScan3D flowanalyzer (One Lambda Inc., CA, USA) using the HLA Fusion software (version no. 4.6). For the evaluation of DSA, beads with raw MFI values > 1,000 were considered to be positive. All sera were pretreated with EDTA in a validated laboratory procedure.

### Statistical Analysis

Statistical analysis was performed using GraphPad Prism 5, Version 5.03 (GraphPad Software, Inc., CA, USA) and IBM SPSS Statistics, Version 24 (International Business Machines Corp., NY, USA). Means and SDs or medians with min and max were used to describe continuous variables. Categorical variables are expressed as *n* and a percentage of the total. Survival analysis was performed by the Kaplan–Meier method, and with differences between groups compared using the log-rank test. The Kaplan–Meier curve was also used to express the ABMR-free interval, defined as the time between transplantation and biopsy-proven active ABMR. Death-censored allograft survival rates are also reported in this study. Univariable and multivariable Cox regression models were used to predict the odds of ABMR, the latter adjusted for applied desensitization treatment and all variables from univariable analysis with *p* < 0.01 (peak panel reactive antibodies (PRA), retransplantation, immunodominant DSA MFI, and DSA number). The area under the curve (AUC) for univariable and multivariable Cox regression was calculated using 10-fold cross-validation (14). A *p-*value of < 0.05 was considered statistically significant.

## Results

### Baseline Characteristics

The study population consisted of 108 deceased donor allograft recipients subjected to peritransplant desensitization. Five living donor transplant recipients who underwent desensitization ahead of scheduled transplantation were excluded from analysis (Figure 1). The baseline characteristics of the study population are given in Table 1. Pretransplant immunological characteristics of recipients are shown in Tables 2, 3. The majority of recipients had anti-HLA class I DSA (*n* = 75, 69%) whereas 13 (12%) of the recipients had class II DSA. Twenty patients (18.5%) had both DSA class I and class II DSA (Table 2). Pretransplant FCXM positivity was identified in 35 recipients (32.4%). Isolated T-cell FCXM positivity was observed in 3 out of 35 (8.6%), isolated B-cell FCXM positivity in 16 out of 35 (45.7%) and both T-cell and B-cell FCXM were observed in 16 out of 35 patients (45.7%), respectively. Sixty patients (55.6%) had only 1 preformed DSA, 37 (34.2%) had 2 DSAs, and 11 (10.2%) had 2–5 DSAs.

**Table 1:** Baseline demographic and clinical characteristics of the study population.

**Patient characteristics (*n* = 108)**
**Recipient characteristics**
Age, years, median [min, max]	53 [23,79]
Sex male, *n (%)*	54 (50%)
**ESRD causes**
Glomerulonephritis, ns, *n (%)*	48 (44.4%)
Interstitial nephropathy, *n (%)*	27 (25%)
Polycystic kidney disease, *n (%)*	11 (10.2%)
Hypertension, *n (%)*	7 (0.06%)
Other, *n (%)*	12 (11.1%)
Undetermined, *n (%)*	3 (0.03%)
Dialysis vintage, months, median [min, max]	40 [0,137]
**Type of dialysis treatment**
Haemodialysis, *n (%)*	86 (79.6%)
Peritoneal dialysis, *n (%)*	11 (10.2%)
Combination of HD and PD, *n (%)*	9 (8.3%)
Preemptive transplantation, *n (%)*	2 (1.9%)
**Donor characteristics**
Age, years, median [min, max]	52 [1,80]
Sex male, *n (%)*	62 (57.4%)
**Donor type**
Deceased donor, *n (%)*	108 (100%)
ECD donor, *n (%)*	43 (39.8%)
**Transplant baseline characteristics**
Retransplantation, *n (%)*	65 (60.2%)
1 prior graft, *n (%)*	47 (43.5%)
2 prior graft, *n (%)*	15 (13.9%)
3 prior graft, *n (%)*	3 (2.8%)
Cold ischemia time, hour, median [min, max]	16 [4,28]
DGF^a^, *n (%)*	25 (23%)
**Induction and desensitization**
IA/PP	88 (81.5%)
IVIg	99 (91.7%)
Rituximab	13 (12%)
ATG	106 (98.1%)
Basiliximab	2 (1.6%)

*Data are presented as median [min, max] or number (%). ESRD, End-stage Renal Disease; HD, Haemodialysis; PD, Peritoneal Dialysis; ECD, Extended Criteria Donor; DGF, Delayed Graft Function; IA, Immunoadsorption; PP, Plasmapheresis; IVIg, Intravenous Immunoglobulins; ATG, Anti-Thymocyte Globuline*.

a *Delayed graft function was defined as the need for dialysis during the first-week post-transplant*.

**Table 2:** Immunological characteristics of human leukocyte antigen incompatible kidney transplant recipients.

**Immunological status**
PRA last, %, mean [SD]	21.3 [26.4]
PRA max, %, median [min, max]	31 [0,100]
HLA mismatch, median [min, max]	4 [0,6]
FCXM positivity, *n (%)*	35 (32.4%)
**Pretransplant DSA**
Immunodominant class I	75 (69.4%)
Immunodominant class II	13 (12%)
Both DSA classes I and II	20 (18.5%)
Immunodominant DSA MFI, median [min, max]	3,344 [1,036, 20,793]
Number of DSA, median [min, max]	1 [1,5]

*Data are presented as mean [standard deviation (SD)] or median [min, max] or n (%). PRA, Panel Reactive Antibodies; HLA, Human Leukocyte Antigen; FCXM, Flow Cytometry Crossmatch; DSA, Donor-specific Antibodies; MFI, Mean Fluorescent Intensity*.

**Table 3:** Pretransplant immunological characteristics of recipients receiving desensitization.

**Immunological status**	**IVIg (*n* = 14)**	**PP/IVIg (*n* = 74)**	**PP/IVIg/RTX (*n* = 14)**	***p*-value**
PRA max, %, mean [SD]	24 [27.9]	41.6 [31.6]	45.8 [32.9]	0.0997
HLA mismatch, mean [SD]	3.2 [0.7]	3.6 [1.4]	3.7 [1.1]	0.5077
FCXM positivity, *n (%)*	0 (0)	24 (32.4)	11 (78.6)	**<0.0001**
MFI, median [IQR]	1,948 [1,194, 2,727]	3,477 [2,150, 5,491]	5,872 [4,996, 10,938]	**<0.0001**
Number of DSA, mean [SD]	1.3 [0.5]	1.5 [0.7]	2.4 [1.4]	**0.0227**

*Data are presented as mean [SD], median [IQR] or number (%). PRA, Panel Reactive Antibodies; HLA, Human Leukocyte Antigen; FCXM, Flow Cytometry Crossmatch; MFI, Mean Fluorescence Intensity; DSA, Donor-specific Antibodies; SD, Standard Deviation; IQR, interquartile range. Bold values indicates statistically significant*.

Two patients had no documented sensitizing events. About 63/108 (58.3%) recipients experienced previous transplantation. A total of 69/108 (63.9%) patients had received the previous transfusion. About 46 of 54 (85.2%) women were previously pregnant.

### Risk of ABMR

Biopsy-proven ABMR (active or chronic active) in indication or protocol biopsies was found in 38 out of 108 patients (35%) within the first 3 years posttransplant. The median time until the first ABMR occurrence was 11 days [min 5, max 1,078]. ABMR rates were highest among patients with immunodominant DSA MFI > 5,000 vs. <5,000 [18 out of 33 (54.5%) vs. 20 out of 75 (26.6%), p = 0.01]. ABMR incidence was higher among patients with a positive vs. negative FCXM [26 out of 35 (74.3%) vs. 12 out of 73 (16.4%), *p* < 0.001], in patients with retransplantation vs. first transplantation [27 out of 63 (42.8%) vs. 11 out of 45 (24.4%), *p* = 0.05] and in patients with total DSA number > 2 vs. those with DSA number ≤ 2 [7 out of 11 (63.6%) vs. 31 out of 97 (32%), p = 0.04].

**Figure 2:**
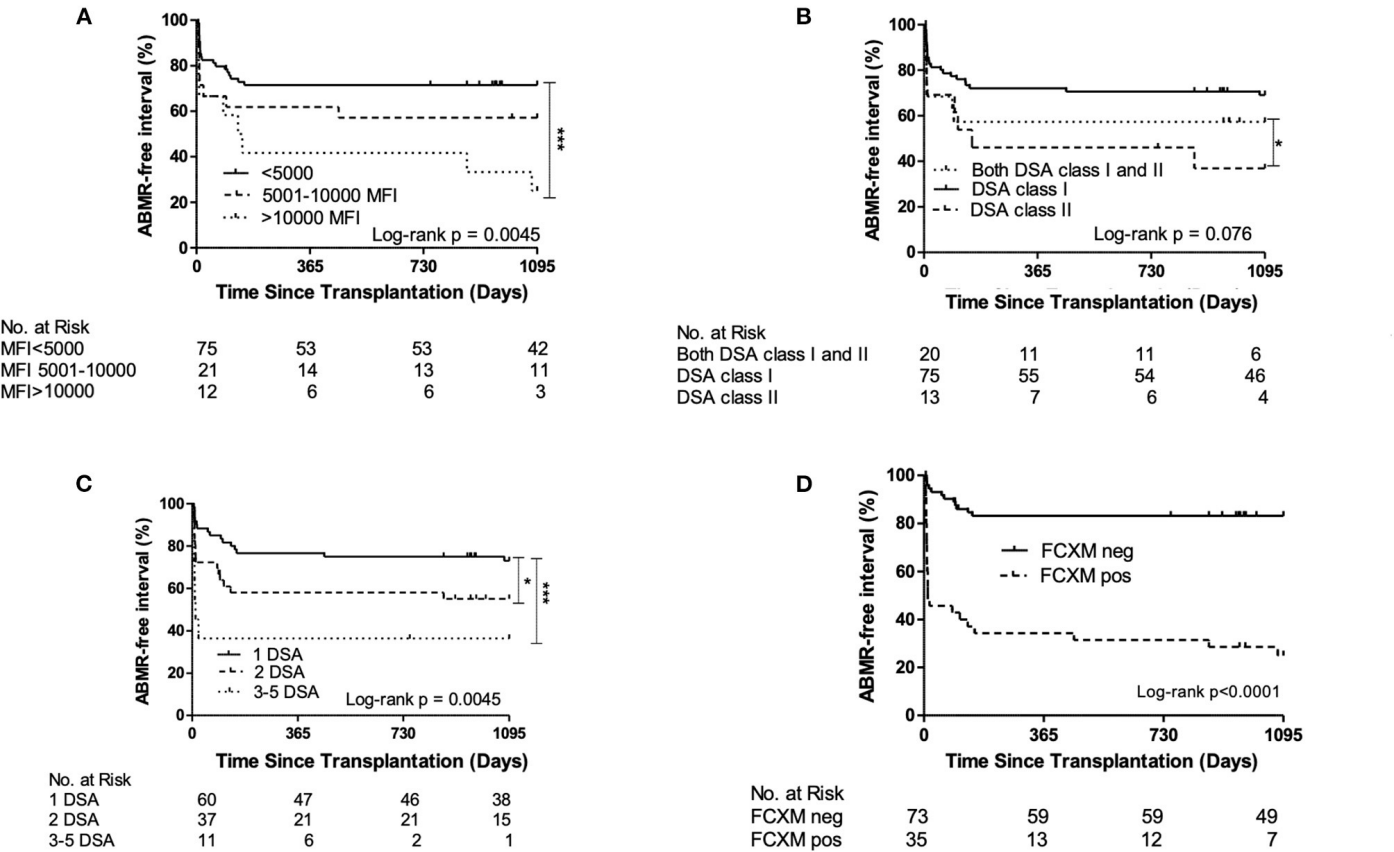
Three-year death-censored antibody-mediated rejection (ABMR)-free interval displaying significantly higher incidence of ABMR in recipients with **(A)** donor-specific antibodies _(DSA) immunodominant mean fluorescence intensity (MFI) > 10,000, **(B)** DSA class II **(C)** higher DSA number and **(D)** patients with positive flow cytometry crossmatch (FCXM). *p < 0.05; ***p < 0.001.

Three-year death-censored ABMR-free interval showed significantly shorter ABMR-free interval in patients with immunodominant MFI > 10,000 (log-rank *p* = 0.0045), with a higher number of DSAs (log-rank *p* = 0.0045) and in patients with pretransplant FCXM positivity (log-rank *p* < 0.0001, Figure 2).

In univariable Cox regression analysis, FCXM positivity (HR = 6.5, *p* < 0.001), retransplantation status (HR = 3.13, *p* = 0.003), recipient gender (HR = 2.1, *p* = 0.026), immunodominant DSA MFI (HR = 1.99, *p* < 0.001), DSA number (HR = 1.6, *p* < 0.001), and peak PRA (HR = 1.02, *p* < 0.001) were found to be associated with ABMR occurrence (Table 4).

**Table 4:** Risk factors of antibody-mediated rejection (ABMR) occurrence in donor-specific antibody plus patients in univariable and multivariable Cox regression model.

	**Univariable Cox regression**			**Multivariable Cox regression**		
	***p*-value**	**HR**	**95% *CI***	***p*-value**	**HR**	**95% *CI***
Recipient age, years	0.025	0.97	0.95–1.00			
Recipient gender, male	0.017	2.26	1.15–4.42			
Donor age, years	0.957	1.00	0.98–1.02			
Dialysis vintage, months	0.128	1.01	1.00–1.02			
Cold ischemia, hours	0.259	1.05	0.96–1.15			
Peak PRA	**<0.001**	**1.02**	1.01–1.03	0.139	1.01	0.99–1.02
HLA mismatch	0.178	1.18	0.93–1.51			
Retransplantation	**0.002**	**3.51**	1.60–7.66	0.177	1.87	0.75–4.63
Immunodominant DSA MFI (increase for each 5,000 MFI)	**<0.001**	**2.05**	1.44–2.92	0.870	0.96	0.61–1.52
DSA number	**0.001**	**1.62**	1.22–2.16	**0.025**	**1.52**	1.05–2.18
Both DSA classes I and II	0.363	1.44	0.66–3.14			
FCXM positivity	**0.000**	**7.31**	3.67–14.58	**<0.001**	**5.47**	2.22–13.49
Rituximab	**0.002**	**3.20**	1.51–6.79	0.891	1.06	0.48–2.34
IA/PP	0.122	2.27	0.80–6.39	0.373	0.56	0.15–2.02
IVIg	0.370	1.92	0.46–7.97	0.506	0.58	0.11–2.92
DSA DQ^a^	**<0.001**	**3.32**	1.67–6.59			

*To final model, all significant variables from univariable Cox regression were entered and the model was adjusted to desensitization protocol. IA, Immunoadsorption; PP, Plasmapheresis; IVIg, Intravenous Immunoglobulins, PRA, Panel Reactive Antibodies; HLA, Human Leukocyte Antigen; FCXM, Flow Cytometry Crossmatch; MFI, Mean Fluorescence Intensity; DSA, Donor-specific Antibodies*.

a *Subgroup analysis from available data (n = 40), not included in the multivariate analysis model. Bold values indicates statistically significant*.

The risk of ABMR in pretransplant positive FCXM remained significant also for patients with class I DSA positivity (HR = 9.63, 95% *CI* = 3.89–22.53, *p* < 0.001) and for those with MFI <5,000 (HR = 6.7, 95% *CI* = 2.75–16.32, *p* < 0.001).

In multivariable Cox regression model adjusted for desensitization regimen, associations remained significant for FCXM positivity (HR = 4.6, *p* = 0.001) and DSA number (HR = 1.47, *p* = 0.039) (Table 3). Multivariable Cox model increased mean AUC (calculated in 10-fold crossvalidation) for pretransplant FCXM positivity from 0.7 to 0.8 (Table 5).

**Table 5:** Area under curve for uni- and multi-variable Cox regression for ABMR risk in 10-fold crossvalidation.

	**Mean AUC**	**SD**
**Univariable Cox model**		
FCXM positivity	0.729	0.04
Peak PRA	0.668	0.02
Immunodominant DSA MFI	0.618	0.03
Retransplantation	0.626	0.04
Total DSA number	0.583	0.04
**Multivariable Cox model**		
(FCXM positivity, peak PRA, Immunodominant DSA MFI, retransplantation, total DSA number adjusted for desensitization treatment)	0.810	0.03

*AUC, Area Under Curve; SD, Standard Deviation; PRA, Panel Reactive Antibodies; FCXM, Flow Cytometry Crossmatch; MFI, Mean Fluorescence Intensity; DSA, Donor-specific Antibodies*.

**Figure 3:**
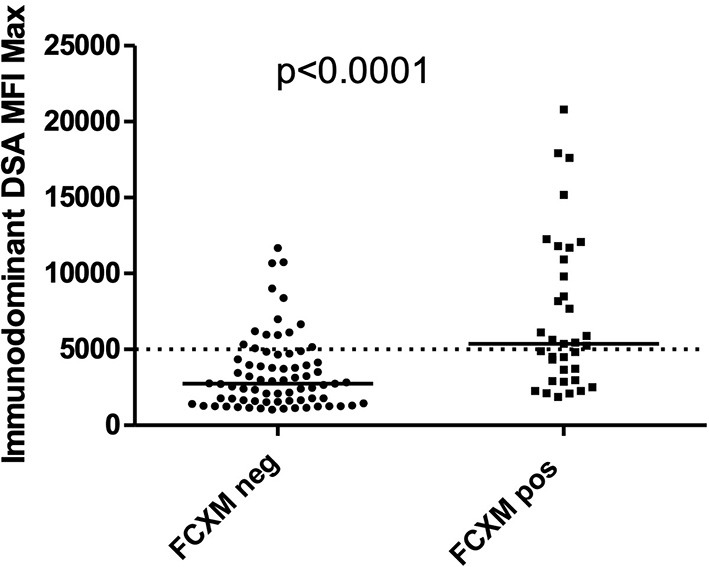
Patients with FCXM positive have significantly higher immunodominant DSA MFI compared to patients with FCXM negative. Nevertheless, 14 out of 73 (20%) of FXCM negative and 19 out of 35 (54%) patients with FCXM positive had immunodominant donor-specific antibody mean fluorescence intensity (DSA MFI) > 5,000, 5 out of 35 (14%) patients with FCXM positive had immunodominant DSA MFI <2,500.

Flow cytometry crossmatch-positive patients had significantly higher immunodominant DSA MFI (Figure 3, *p* < 0.0001). This correlation was, however, not perfect (15, 16) as 15 out of 75 (20%) of FXCM-negative subjects had immunodominant DSA MFI > 5,000 (3 subjects with DSA-MFI > 10,000). Similarly, in FCXM-positive patients, 4 out of 33 (12%) had immunodominant DSA-MFI <2,500.

### Graft Survival

Three-year death-censored graft survival was shorter in patients with a positive FCXM (*p* = 0.011) and those with both DSA classes I and II (*p* = 0.04) or a higher number of DSA (*p* < 0.001) (Supplementary Figure 1).

## Discussion

Kidney transplantation across the HLA barrier is associated with an inferior allograft outcome. It is widely acknowledged that the presence of preformed DSA before transplantation increases the probability of ABMR occurrence (12–15). Strategies, namely, peritransplant desensitization, were implemented in many centers to prevent such an adverse outcome (17–19).

In this retrospective single-center analysis, we evaluated the potential of several clinical and immunological risk factors to predict ABMR in patients who had received deceased donor kidney allograft and in whom the peritransplant desensitization was applied due to the current presence of donor-specific antibodies. We found that pretransplant FXCM and the number of DSAs, but not MFI, are the most reliable tools for ABMR prediction.

It has been widely accepted that the prognostic value of DSA is limited (20, 21). First, Luminex-based DSA determination shows HLA antibody specificity to antigen but no information can be provided in regardsto epitope specificity (22, 23). Second, the Luminex method is unsuitable for exact value measurement because its MFI values are semiquantitative, not presenting the exact titer of antibodies; furthermore, the prozone effect must be taken into account, non-anti-HLA antibodies may interfere with the beads, and the previously mentioned epitopes may be shared between the beads (24, 25).

In many centers, the FCXM assessment is not implemented as a 24/7 service, and therefore, there is a lack of information on whether FCXM outperforms DSA-based risk stratification. Thus, studies on FCXM are not consistent in respect of graft outcomes (10, 26, 27). Our data are similar to Couzi et al. (10), where higher rejection occurrence in patients with both DSA and FCXM positivity prior to transplantation was observed.

Our data from protocol biopsies suggest future long-term outcomes to be inferior as patients with positive pretransplant FCXM exhibit frequent subclinical rejections in 3-month protocol biopsies. However, it is well known that sensitized patients who have undergone desensitization and HLA incompatible living donor kidney transplantation have substantial survival benefits compared to those patients who did not undergo transplantation and those who waited for transplants from deceased donors (28). However, the overall incidence of ABMR in those patients was high when 24 out of 267 patients developed severe oliguric early ABMR treated with eculizumab or splenectomy and transplant glomerulopathy occurred later (29).

Data on HLA incompatible deceased donor kidney transplantation are scarce. In their pioneer work, the Vienna group evaluated the outcomes of 101 HLA incompatible kidney transplantation (9) who had undergone IA-based desensitization prior and after transplantation along with rabbit anti-thymocyte globulin. Most DSA+ patients had a negative CDC XM already before IA. Three-year death-censored graft survival in DSA+ patients was 79 and 33% of patients experienced ABMR. The authors described only a trend toward higher ABMR rates in positive baseline CDCXM while those patients with DSA MFI >15,000 experienced ABMR in 71%. Similarly to our study, Amrouche et al. (30) described in 95 patients, who received similar posttransplant desensitization due to DSA MFI levels >3,000 while negative complement-dependent cytotoxicity-negative crossmatch, satisfactory long-term outcomes: the 3-year death-censored allograft survival rates were 91%, and recipient survival rates were 93%, respectively. Those data are similar to our FCXM-negative cohort. Of note, the incidence of ABMR in the Amrouche study remained high and was detected in 32% of recipients which is similar to our FCXM negative cohort. Contrary to expectations, in our study, the FCXM positive cohort presented with far poorer outcomes in terms of ABMR incidence and 3-year graft outcomes. Therefore, it is likely that our HLA incompatible cohort was at a far higher risk in comparison with others. This fact also points out the necessity of more advanced risk stratification ahead of transplantation which may allow successful kidney transplantation even in patients with DSA MFI over 5,000. Our study shows the association of pretransplant FCXM with ABMR also in patients with MFI <5,000 or with class I DSA. Therefore, it is likely that pretransplant FCXM positivity outperforms any DSA level from historical sera.

Based on the presented data, we have already modified a pre-transplant risk assessment in our center. In all kidney transplant recipients with present preformed DSA, the positive FCXM prior to transplant represents a veto for transplantation. Unpublished data suggest on far lower incidence of acute ABMR when this approach was implemented. It is, however, also likely that those patients with repeated positivity of FCXM prior to transplant are being trapped on the waiting list for a significant period of time, although the general waiting time to transplant is much shorter in comparison to other countries (31). Of note, described the poor outcome of HLA incompatible transplantation with pretransplant FCXM positivity justifies longer waiting time for more compatible donors as there is a lack of available organs and better allocation would finally increase the patient-years with functioning grafts. Some of those sensitized patients may also find their compatible donor in the case of kidney pair donation when a living donor is available (32).

The limitation of this study is the lack of DSA DP and DQ assessment, as donor DP and DQ typing were not available in the whole cohort.

In conclusion, our data suggest that pretransplant FCXM and DSA number, but not DSA MFI, are independent predictors of ABMR in patients who received peritransplant desensitization. We, therefore, suggest the implementation of FCXM assessment in a daily routine ahead of DSA positive deceased donor kidney transplantation when peritransplant desensitization is planned.

## Data Availability Statement

The raw data supporting the conclusions of this article will be made available by the authors, without undue reservation.

## Ethics Statement

The studies involving human participants were reviewed and approved by the Ethics Committee of the Institute for Clinical and Experimental Medicine and Thomayer Hospital (listed as No. A-19-24). The patients/participants provided their written informed consent to participate in this study.

## Author Contributions

OV and GB designed the research. KO, GB, and OV wrote the manuscript. KO and PH collected the data. KO, OV, JS, AS, JM, and TM performed the research. KK provided the FCXM sera analysis. KO, PH, and JK participated in the data analysis and OV supervised the research. All authors contributed to the article and approved the submitted version.

## Funding

This study was supported by the Ministry of Health of the Czech Republic MZO 00023001 and by the Ministry of Health of the Czech Republic under grants NV19-06-00031 and NU21-06-00021. The authors wish to thank the staff of Immunogenetics Laboratory and Transplant Laboratory for their valuable help with FCXMs evaluation. They are also grateful to Michael Fitzgerald for the English correction.

## Conflict of Interest

The authors declare that the research was conducted in the absence of any commercial or financial relationships that could be construed as a potential conflict of interest.

## Publisher's Note

All claims expressed in this article are solely those of the authors and do not necessarily represent those of their affiliated organizations, or those of the publisher, the editors and the reviewers. Any product that may be evaluated in this article, or claim that may be made by its manufacturer, is not guaranteed or endorsed by the publisher.

## Abbreviations

ABMR: antibody-mediated rejection
CDC XM: complement-dependent cytotoxicity crossmatch
DD: deceased donor
DGF: delayed graft function
DSA: donor-specific antibody
eGFR: estimated glomerular filtration
FCXM: flow cytometry crossmatch
HD: haemodialysis
HLA: human leukocyte antigen
HLAi: HLA incompatible
IVIg: intravenous immunoglobulin
MFI: mean fluorescence intensity
MMF: mycophenolate mofetil
MVA: multivariate analysis
PD: peritoneal dialysis
PRA: panel reactive antibodies
rATG: rabbit anti-thymocyte globulin
RTX: rituximab
FITC: fluorescein.
