# Supplementary material for: Predictive Potential of Flow Cytometry Crossmatching in Deceased Donor Kidney Transplant Recipients Subjected to Peritransplant Desensitization
Source: Front Med (Lausanne). 2021 Dec 14;8:780636. doi: 10.3389/fmed.2021.780636 (PMC8712553; doi:10.3389/fmed.2021.780636)
Supplement: Supplementary file 1 [file Data_Sheet_1.docx]

Supplementary Material

## Supplementary Figures


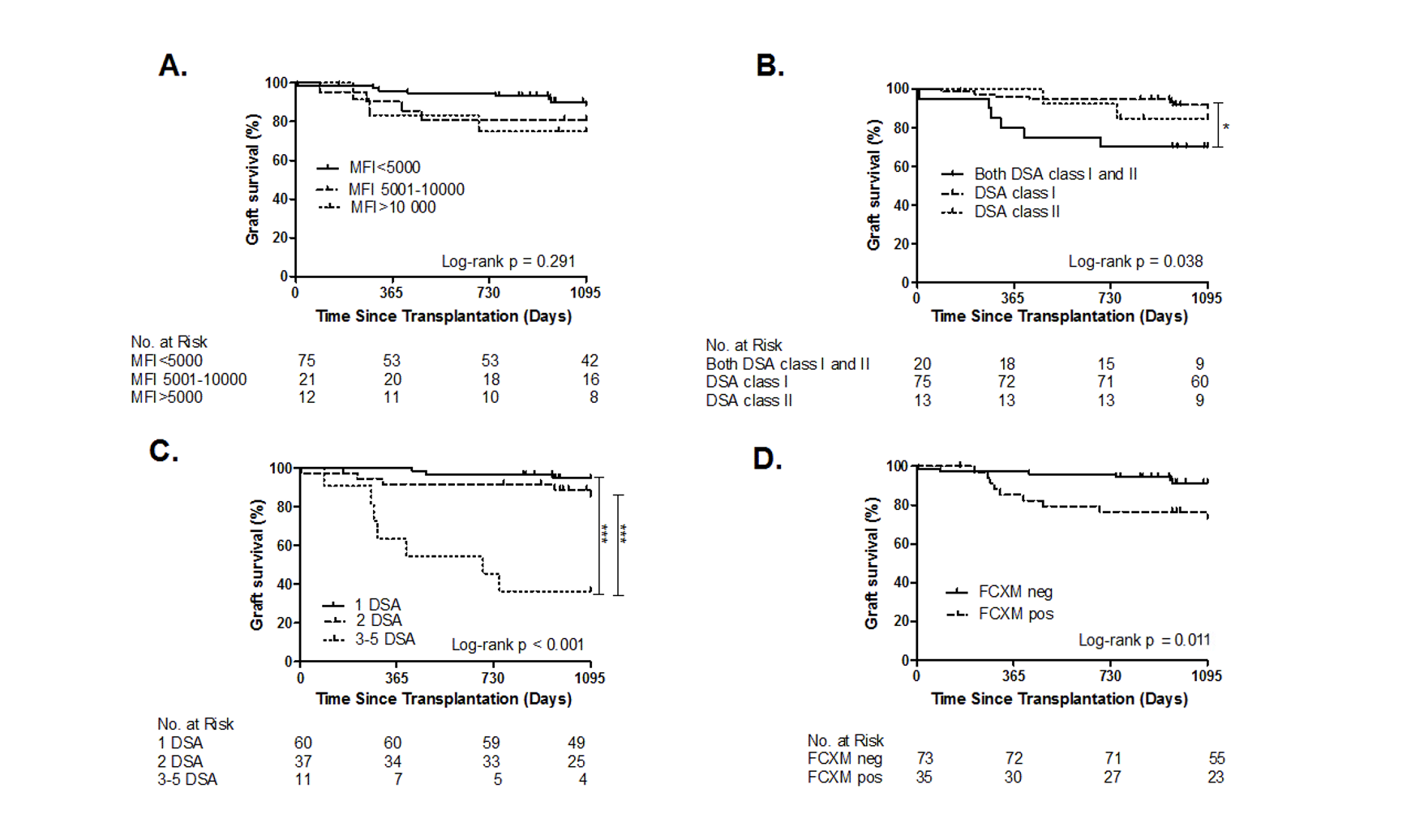
**Supplemental Figure S1:** Three-year death-censored graft survival displaying non-significantly worse graft survival in patients with higher immunodominant max MFI (A) and significantly shorter survival in recipients with B. DSA class II C. higher DSA number and D. patients with positive FCXM.
